# Supplementary material for: VPS34 K29/K48 branched ubiquitination governed by UBE3C and TRABID regulates autophagy, proteostasis and liver metabolism
Source: Nat Commun. 2021 Feb 26;12:1322. doi: 10.1038/s41467-021-21715-1 (PMC7910580; doi:10.1038/s41467-021-21715-1)
Supplement: Supplementary file 1 — Supplementary Information [file 41467_2021_21715_MOESM1_ESM.pdf]

## Supplementary Information

# VPS34 K29/K48 branched ubiquitination governed by UBE3C and TRABID regulates autophagy, proteostasis and liver metabolism

## Supplementary Figure

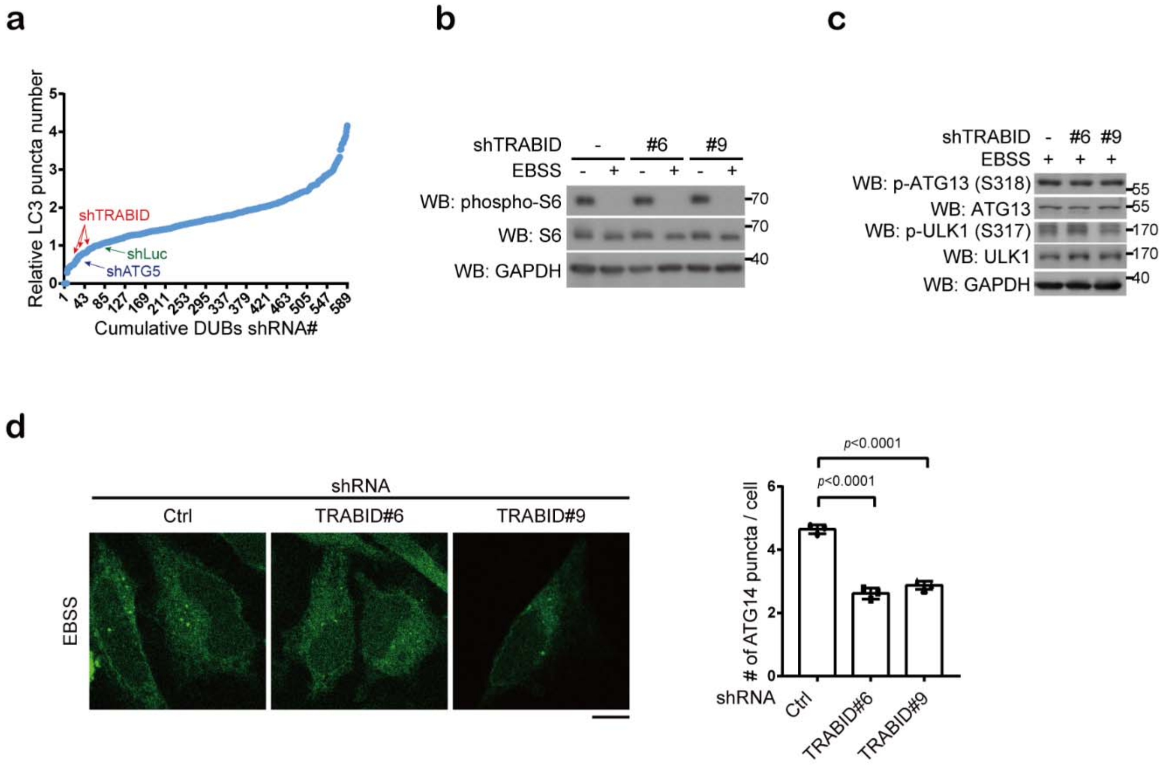


**Supplementary Fig. 1. TRABID regulates autophagy without affecting mTOR, AMPK and ULK1 activities.**

**a** Screen of DUBs for regulating autophagy. Scores of numbers of LC3 puncta for 587 shRNAs representing 92 DUBs, together with Luc shRNA and ATG5 shRNA are plotted. The positions of three TRABID shRNAs, Luc shRNA (negative control), and ATG5 shRNA (positive control) are indicated by arrows. **b** Western blot analysis of S6 phosphorylation in HeLa cells stably expressing TRABID shRNAs cultured in full medium or EBSS for 2 h. **c** Western blot analysis of ULK1 and ATG13 phosphorylation in HeLa cells expressing TRABID shRNAs cultured in EBSS for 2 h.

**d** HeLa cells expressing TRABID shRNAs were transfected with GFP-ATG14 and cultured in EBSS for 2 h. GFP-ATG14 puncta were analyzed by confocal microscopy. Representative images are shown on the left and quantitative data are on the right. Bar, 10 μm. Data are mean ± SD (n=3 independent experiments, and 30 cells per group per experiment were counted). *P* values are determined by one-way ANOVA with Tukey's post hoc test.

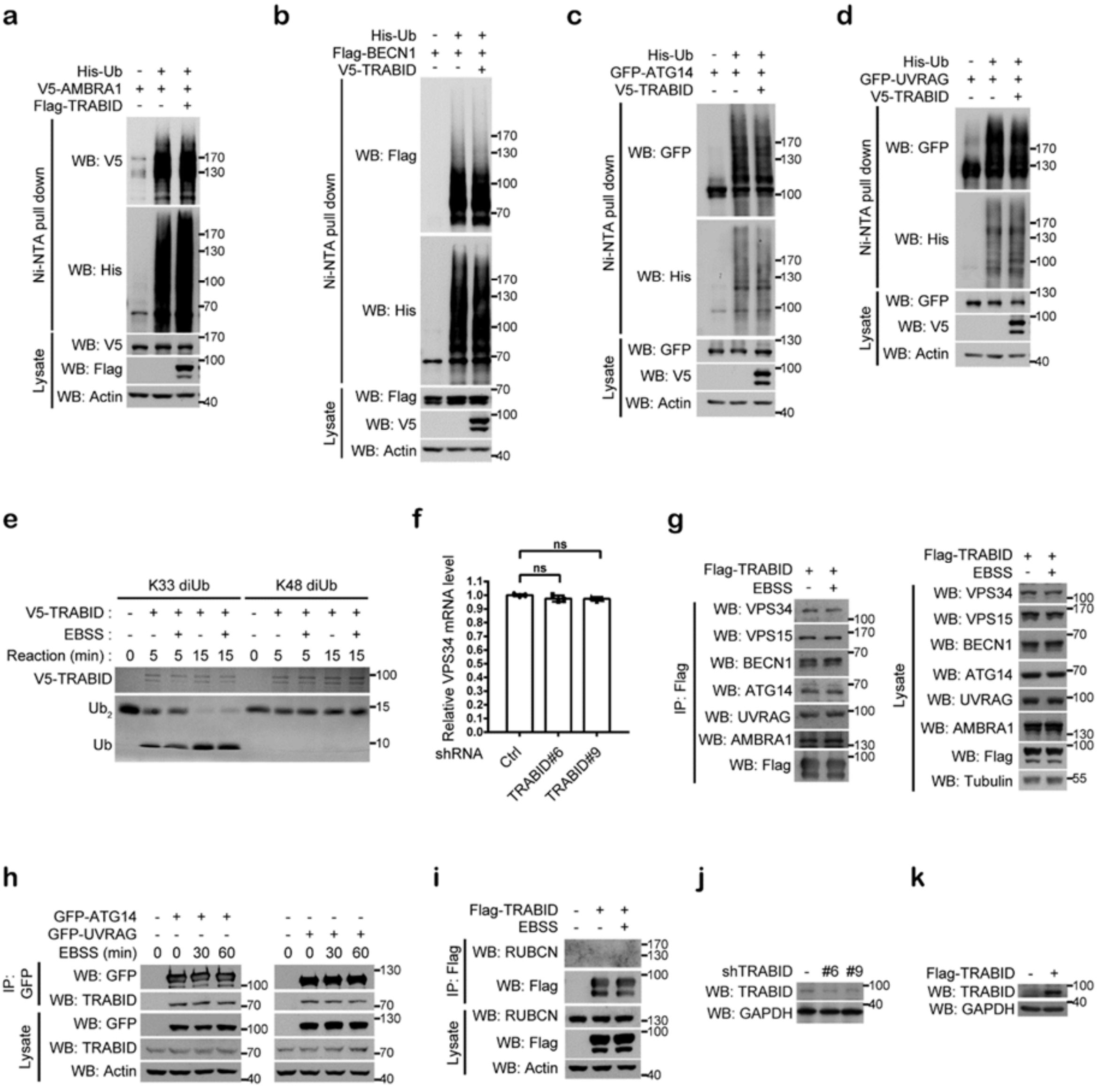


**Supplementary Fig. 2. TRABID is associated with VPS34 complex I and II, but cannot affect the ubiquitination of AMBRA1, Beclin-1, ATG14 and UVRAG.**

**a-d** Analysis of AMBRA1 (**a**), Beclin-1 (**b**), ATG14 (**c**), and UVRAG (**d**) ubiquitination in 293T cells transfected with indicated constructs. The ubiquitinated proteins were pulled down by Ni-NTA agarose under denaturing conditions and analyzed by Western blot with indicated antibodies. **e** In vitro disassembly of K33-linked and K48-linked diubiquitin chain by TRABID purified from cells cultured in full medium or EBSS. The reaction was proceeded for indicated time periods and then analyzed by

SDS-PAGE and Coomassie blue staining. **f** *VPS34* mRNA expression assayed by RT-qPCR in HeLa cells expressing control and TRABID shRNAs. Data are mean ± SD (n=3 independent experiments, ns: not significant by one-way ANOVA with Tukey's post hoc test). **g** Immunoprecipitation analysis of the interaction between Flag-TRABID and various subunits of the VPS34 complex I and II in 293T cells transfected with Flag-TRABID and cultured in full medium or EBSS for 2 h. **h** Immunoprecipitation analysis of the interaction between TRABID and ATG14 or UVRAG in 293T cells transfected with indicated constructs and cultured in full medium or EBSS for indicated time points. **i** TRABID cannot interact with Rubicon. Immunoprecipitation analysis of the interaction between Flag-TRABID and Rubicon in 293T cells transfected with Flag-TRABID and cultured in full medium or EBSS for 2 h. **j, k** Western blot analysis of TRABID expression in indicated HeLa-RFP-GFP-LC3 derivatives.

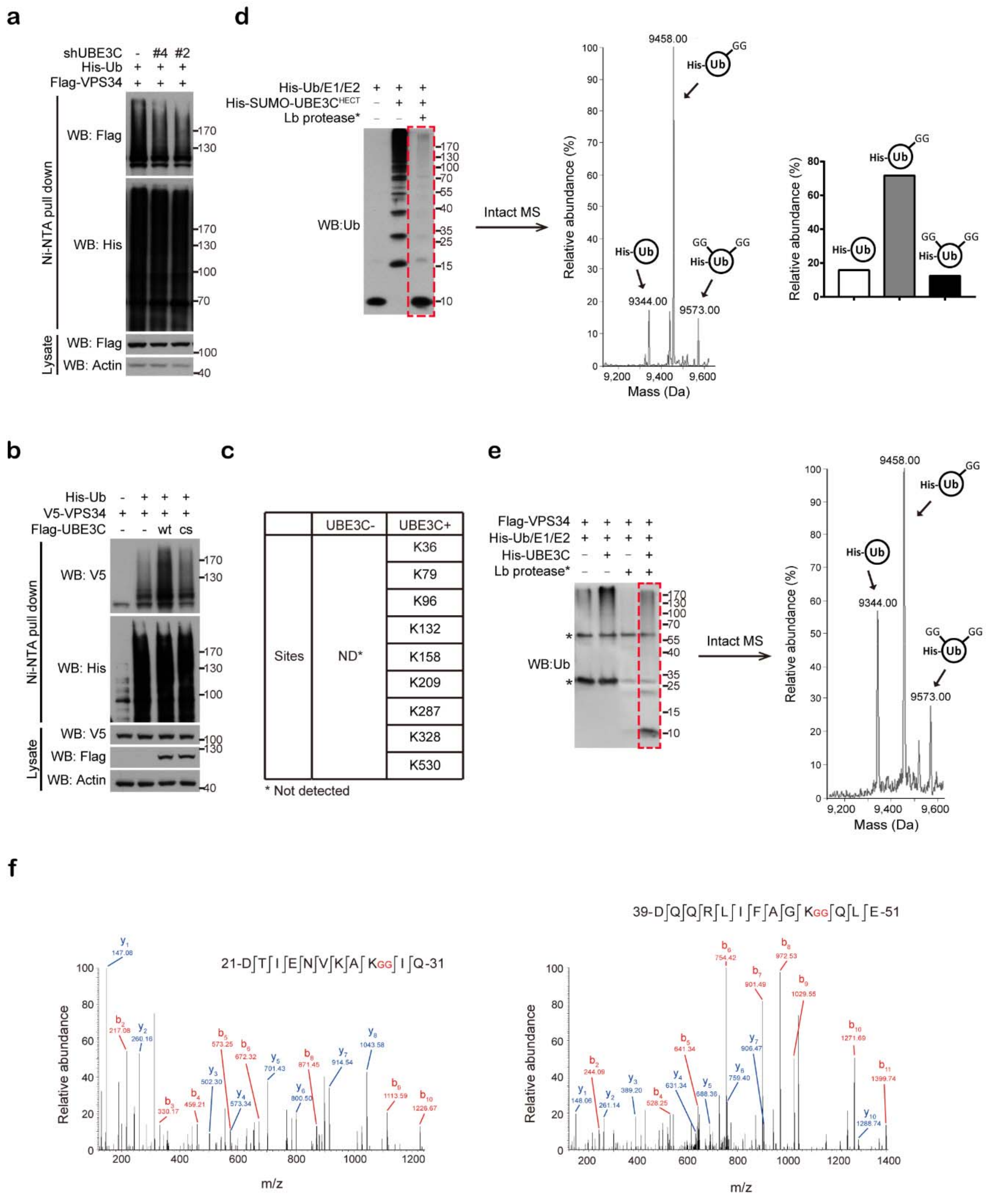


**Supplementary Fig. 3. UBE3C promotes VPS34 branched ubiquitination.**

**a** Analysis of VPS34 ubiquitination in 293T cells stably expressing UBE3C shRNA and transfected with indicated constructs. The ubiquitinated proteins were pulled down by Ni-NTA agarose under denaturing conditions and analyzed by Western blot with antibodies as indicated. **b** Analysis of VPS34 ubiquitination in 293T cells

transfected with indicated constructs. The ubiquitinated proteins were pulled down by Ni-NTA agarose under denaturing conditions and analyzed by Western blot with indicated antibodies. **c** Summary of VPS34 ubiquitination sites identified by LC-MS/MS analysis of VPS34 derived from in vivo ubiquitination assays of UBE3C-transfected (UBE3C+) or untransfected (UBE3C-) 293T cells. **d** UBE3C assembles branched free ubiquitin chains in vitro. (Left panel) Ub-clipping assay of the free ubiquitin chain assembled in vitro by UBE3C$^{HECT}$ (the catalytic domain of UBE3C). (Middle panel) Intact MS analysis of reaction product as shown in lane 3 of left panel. Spectrum was deconvoluted and peaks corresponding to unmodified, single- and double-GG modified ubiquitin are indicated. (Right panel) Quantification of the relative abundance of each ubiquitin species. **e** Assembly of branched ubiquitin chain on VPS34 by UBE3C. (Left panel) Ub-clipping assay of the ubiquitin chain assembled on VPS34. VPS34 bound on beads was ubiquitinated in vitro in the presence or absence of UBE3C. The beads were washed, treated with or without Lb$^{pro*}$ and analyzed by Western blot. Asterisks denote the immunoglobulin heavy and light chains. (Right panel) Intact MS analysis of reaction product as shown in lane 4 of left panel. Spectrum was deconvoluted and peaks corresponding to unmodified, single- and double-GG modified ubiquitin are indicated. **f** Representative tandem mass spectra showing the presence of GG-modification in the K29 (left) and K48 (right) residues of ubiquitin. The clipped ubiquitin monomer derived from an experiment as shown in lane 3 and lane 4 of **e** were analyzed by LC-MS/MS.

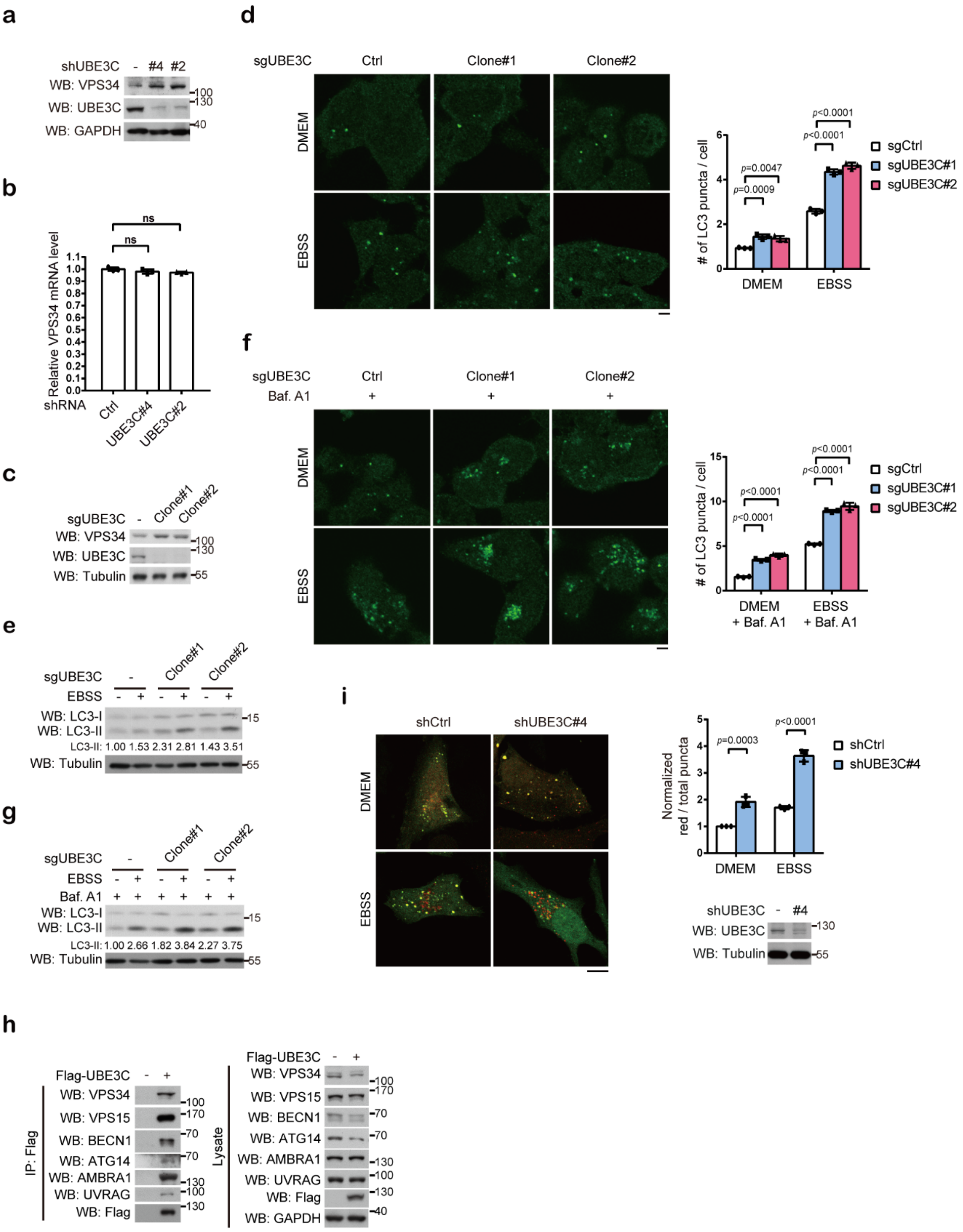


**Supplementary Fig. 4. UBE3C promotes VPS34 degradation to inhibit autophagosome formation and maturation.**

**a, c** Western blot analysis of VPS34 levels in HeLa cells stably expressing UBE3C shRNAs (**a**) or in the two clones of 293T UBE3C KO cells (**c**). **b** *VPS34* mRNA

expression assayed by RT-qPCR in HeLa cells expressing control and UBE3C shRNAs. Data are mean ± SD (n=3 independent experiments, ns: not significant by one-way ANOVA with Tukey's post hoc test). **d**, **f** Immunofluorescence staining of LC3 in 293T UBE3C knockout cells cultured in full medium or EBSS in the absence (**d**) or presence (**f**) of bafilomycin A1 for 2 h. Representative confocal images are shown on the left and quantitative data are on the right. Bar, 10 μm. **e**, **g** Western blot analysis of LC3 in 293T UBE3C knockout cells cultured in full medium or EBSS in the absence (**e**) or presence (**g**) of bafilomycin A1 for 2 h. **h** Immunoprecipitation analysis of the interaction between UBE3C and indicated subunits of VPS34 complex I and II in 293T cells transfected with Flag-UBE3C. **i** Immunofluorescence analysis of autophagosome maturation in indicated HeLa-RFP-GFP-LC3 derivatives cultured in full medium or EBSS for 2 h. Representative images are shown on the left. Bar, 10 μm. The ratios of red puncta to total puncta were quantified and plotted on the right. Data in **d**, **f** and **i** are mean ± SD (n=3 independent experiments, and 30 (for **d**, **f**) or 10 (for **i**) cells per group per experiment were counted). *P* values are determined by two-way ANOVA with Tukey's post hoc test. ns, not significant.

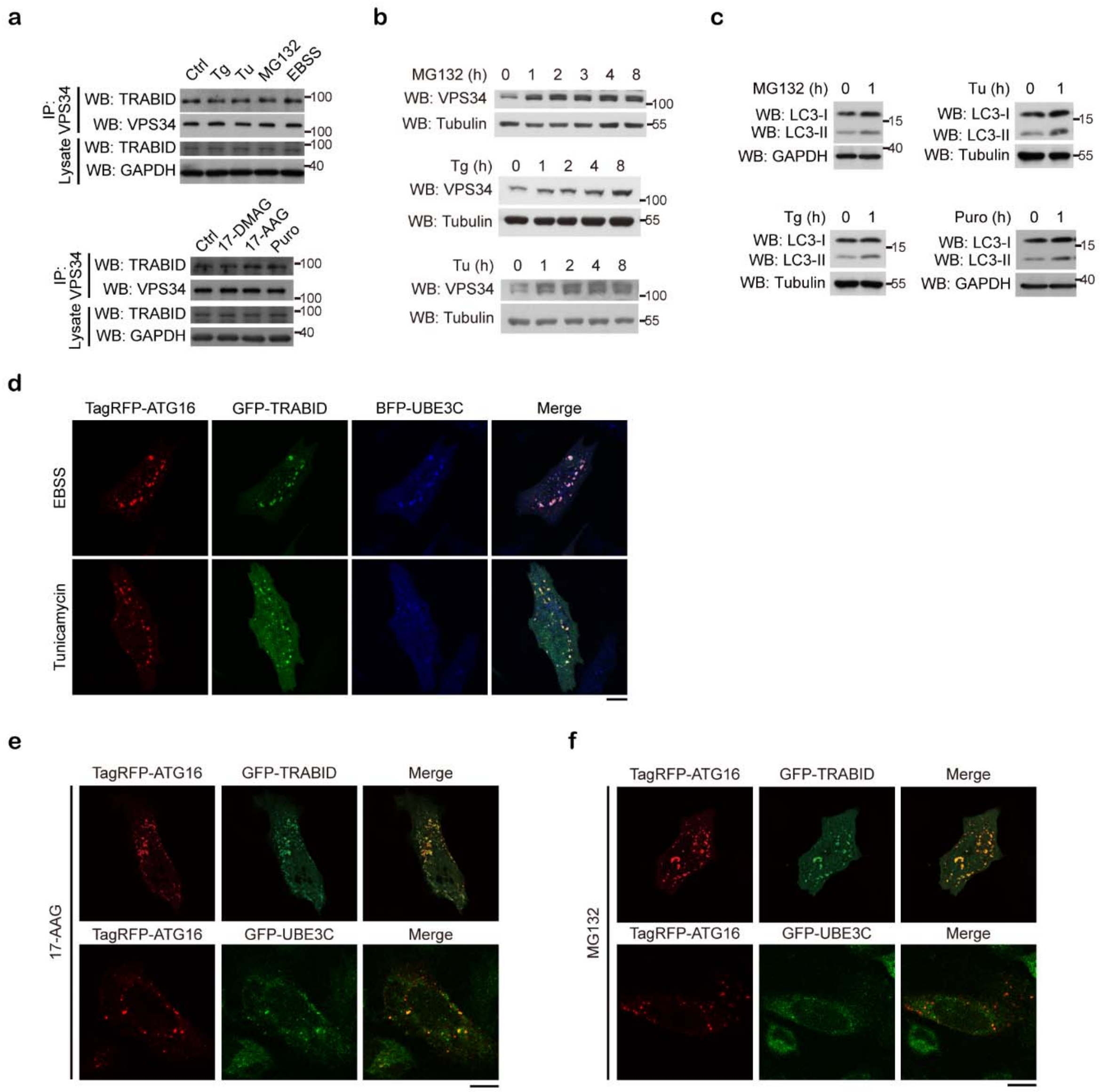


**Supplementary Fig. 5. ER and proteotoxic stresses decrease UBE3C phagophore recruitment to increase VPS34 abundance and autophagy activity.**

**a** Immunoprecipitation analysis of the interaction between endogenous TRABID and VPS34 in 293T cells treated with indicated agents for 1 h. **b** Western blot analysis of VPS34 levels in 293T cells treated with indicated agents for indicated time points. **c** Western blot analysis of LC3 in 293T cells treated with indicated agents for 1 h. **d** Confocal analysis of the colocalization of TagRFP-ATG16 with GFP-TRABID and BFP-UBE3C in triple transfected HeLa cells cultured in EBSS or treated with tunicamycin for 1 h. Bar, 10 $\mu$m. **e**, **f** Confocal analysis of the colocalization of

TagRFP-ATG16 with GFP-TRABID or GFP-UBE3C in transfected HeLa cells treated with 17-AAG (**e**) or MG132 (**f**) for 1 h. Bar, 10 μm.

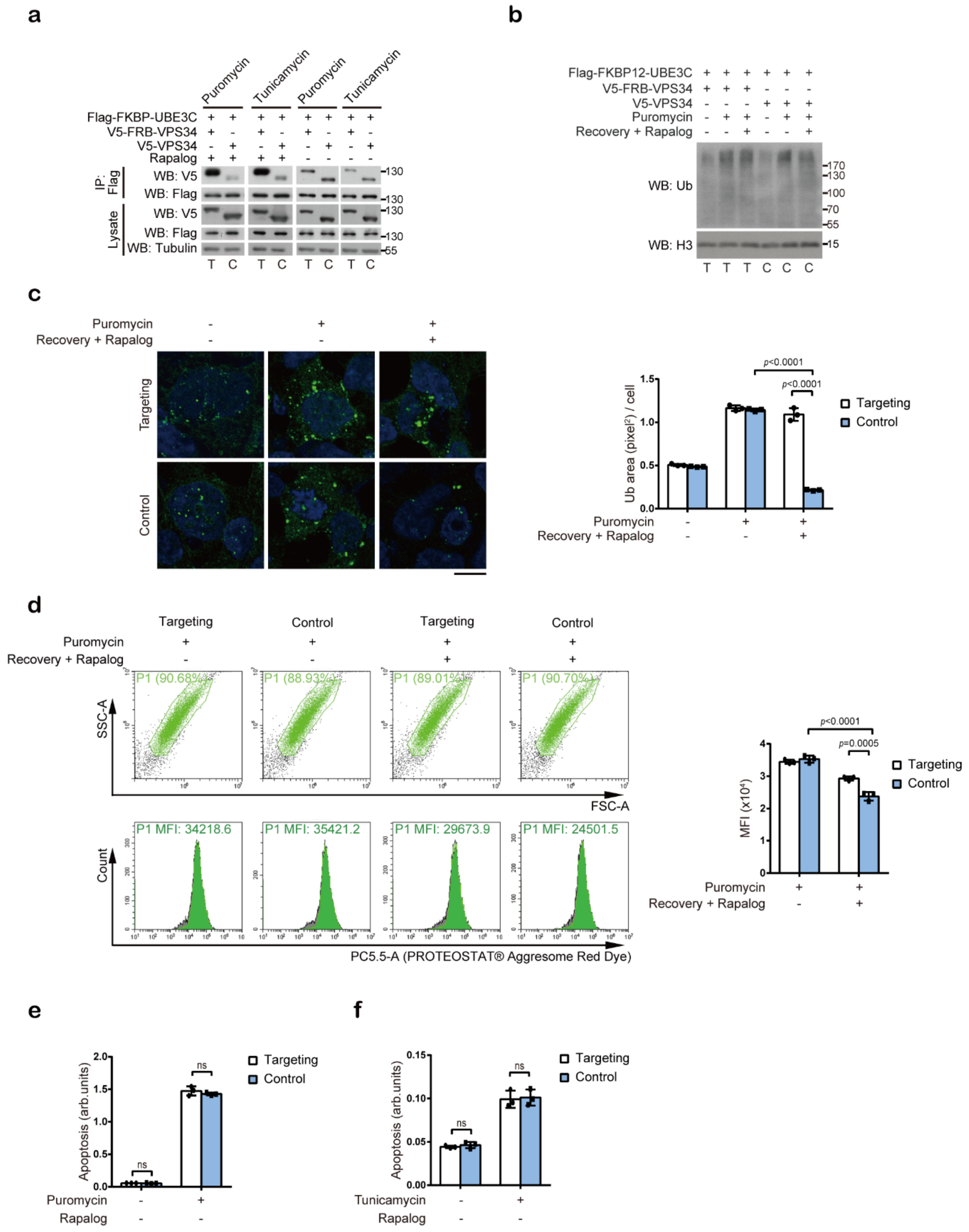


**Supplementary Fig. 6. Enforced association of UBE3C with VPS34 impairs protein aggregate clearance.**

**a** 293T UBE3C KO-derived targeting (T) or control cells (C) as shown in Fig. 6a were

treated with 10 μg/ml puromycin or tunicamycin together with 0.5 μM rapalog for 30 min and analyzed for the interaction between transfected UBE3C and VPS34. **b, c** 293T UBE3C KO-derived targeting or control cells were treated with puromycin for 2 h. After washing out puromycin, cells were treated with rapalog and recovered for 4 h. Cells were then lysed by RIPA buffer and the insoluble fraction was analyzed by Western blot (**b**). Alternatively, cells were analyzed by immunofluorescence staining for ubiquitin (**c**). Representative images are on the left and quantitative data are on the right. Bar, 10 μm. Data are mean ± SD (n=3 independent experiments, and 30 cells per group per experiment were counted). *P* values are determined by two-way ANOVA with Tukey's post hoc test. **d** 293T UBE3C KO-derived targeting or control cells were treated as in **c**, stained by PROTEOSTAT® dye and analyzed by flow cytometry for mean fluorescence intensity (MFI). The representative gating information and MFI values are shown on upper left and lower left panels, respectively, whereas the quantitative data are shown on the right. Data are mean ± SD (n=3 independent experiments). *P* values are determined by two-way ANOVA with Tukey's post hoc test. **e**, **f** 293T UBE3C KO-derived targeting or control cells were treated with puromycin for 3 h (**e**) or tunicamycin for 6 h (**f**) and analyzed for apoptosis. Data are mean ± SD (n=3 independent experiments). *P* values are determined by two-way ANOVA with Tukey's post hoc test, ns, not significant.

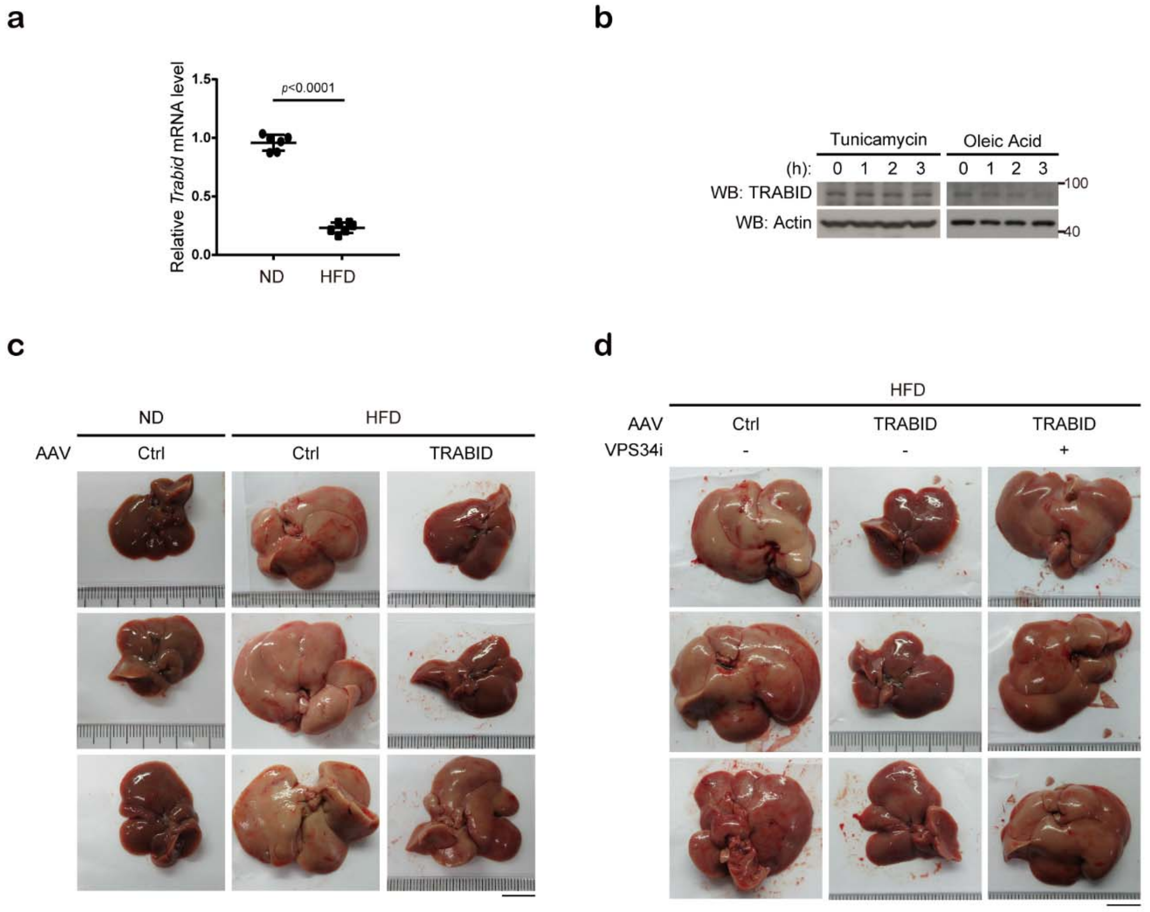


**Supplementary Fig. 7. TRABID is downregulated during NAFLD pathogenesis and plays an inhibitory role in NAFLD through VPS34.**

**a** *Trabid* mRNA expression analyzed by RT-qPCR from livers of indicated mice. Data are mean ± SD from 6 mice. *P* value is determined by unpaired two-sided t test. **b** Western blot analysis of TRABID expression in AML12 cells treated with 100 μM oleic acid or 2 μM tunicamycin for indicated time points. **c, d** TRABID alleviates liver steatosis in NAFLD model mice, which is reversed by VPS34 inhibition. Gross view for the livers taken from indicated mice is shown. Bar, 1 cm.

Table 1: Sequences and sources of shRNAs and sgRNAs

| shRNA | Target sequences | SOURCE (IDENTIFIER) |
|---|---|---|
| Luciferase shRNA | CTTCGAAATGTCCGTTCGGTT | National RNAi Core Facility, Academia Sinica, Taiwan |
| TRABID shRNA#6 | CCATAGAAGCATACAAGTCAT | National RNAi Core Facility, Academia Sinica, Taiwan |
| TRABID shRNA#9 | CAAGGGTGAAATCTTCGTATA | National RNAi Core Facility, Academia Sinica, Taiwan |
| UBE3C shRNA#2 | GTCCTATTTCTATCTCCACTT | National RNAi Core Facility, Academia Sinica, Taiwan |
| UBE3C shRNA#4 | GCAGATAAGCAAGAAGTTCAA | National RNAi Core Facility, Academia Sinica, Taiwan |
| UBE3C sgRNA#1 | CGGCGGCGCTGCCCGCACAT | National RNAi Core Facility, Academia Sinica, Taiwan |
| UBE3C sgRNA#2 | CTGGACTCGGGGCCGAGACT | National RNAi Core Facility, Academia Sinica, Taiwan |
| Pooled UBE3C siRNAs | Not available by vendor | Horizon Discovery (Cat# L-007183-00-0005) |

Table 2: Sequences of qPCR primers

| Gene name | | Sequence (5’ to 3’) |
|---|---|---|
| VPS34 | F | CAAGTTTTTGCAGAAGGGAAGC |
| | R | GAGCTTTGGTGAGCTTGGCA |
| UBE3C | F | TGGCCCCAACCTTACCCTT |
| | R | GCAGCAACCTGCAACAGAG |
| GAPDH | F | TGTTGCCATCAATGACCCCTT |
| | R | CTCCACGACGTACTCAGCG |
| Trabid | F | CCAGACTCTAGTGCAAGACCA |
| | R | GGTCCTACGCTGGGATAAGC |
| Gapdh | F | AGGTCGGTGTGAACGGATTTG |
| | R | TGTAGACCATGTAGTTGAGGTCA |
