# Supplementary material for: VPS34 K29/K48 branched ubiquitination governed by UBE3C and TRABID regulates autophagy, proteostasis and liver metabolism
Source: Nat Commun. 2021 Feb 26;12:1322. doi: 10.1038/s41467-021-21715-1 (PMC7910580; doi:10.1038/s41467-021-21715-1)
Supplement: Supplementary file 5 — Description of Additional Supplementary Files [file 41467_2021_21715_MOESM5_ESM.docx]

Description of Additional Supplementary files

Title: Supplementary data 1

Description: Results of DUB shRNA screen

Title: Supplementary data 2

Description: Antibody details
